# A Trivers-Willard Effect in Contemporary Humans: Male-Biased Sex Ratios among Billionaires

**DOI:** 10.1371/journal.pone.0004195

**Published:** 2009-01-14

**Authors:** Elissa Z. Cameron, Fredrik Dalerum

**Affiliations:** Mammal Research Institute, Department of Zoology and Entomology, University of Pretoria, Pretoria, South Africa; University of Sussex, United Kingdom

## Abstract

**Background:**

Natural selection should favour the ability of mothers to adjust the sex ratio of offspring in relation to the offspring's potential reproductive success. In polygynous species, mothers in good condition would be advantaged by giving birth to more sons. While studies on mammals in general provide support for the hypothesis, studies on humans provide particularly inconsistent results, possibly because the assumptions of the model do not apply.

**Methodology/Principal Findings:**

Here, we take a subset of humans in very good condition: the Forbe's billionaire list. First, we test if the assumptions of the model apply, and show that mothers leave more grandchildren through their sons than through their daughters. We then show that billionaires have 60% sons, which is significantly different from the general population, consistent with our hypothesis. However, women who themselves are billionaires have fewer sons than women having children with billionaires, suggesting that maternal testosterone does not explain the observed variation. Furthermore, paternal masculinity as indexed by achievement, could not explain the variation, since there was no variation in sex ratio between self-made or inherited billionaires.

**Conclusions/Significance:**

Humans in the highest economic bracket leave more grandchildren through sons than through daughters. Therefore, adaptive variation in sex ratios is expected, and human mothers in the highest economic bracket do give birth to more sons, suggesting similar sex ratio manipulation as seen in other mammals.

## Introduction

Natural selection should favour adaptive variation in offspring sex ratio if alterations maximise the offspring's potential reproductive success [Trivers-Willard hypothesis, TWH; 1]. For species where one sex has more variable reproductive success (males in polygynous species), the TWH predicts that 1) a mother with more resources to invest would be advantaged by producing a son, as a successful son would out-compete a successful daughter (constrained to a less variable reproductive rate), and 2) a mother with less resources to invest would be advantaged by producing a daughter, as her daughter would out-reproduce an unsuccessful son. Alternatively, if sons are more costly than daughters, only mothers in good condition could bear this cost [2].

For the predictions of the TWH to apply, the assumptions of the model must be met [3], [4]. Specifically, maternal condition should influence offspring condition, the offspring's condition should endure into adulthood, and any condition advantages should have a greater effect on the more reproductively variable sex [1], males in humans. The first two assumptions hold in humans. There is a strong association between birthweights of mothers and offspring [5], which seems to be largely environmentally determined [6]. Furthermore, birthweight is associated with survival and future reproductive success [7], [8], suggesting that condition advantages endure into adulthood. However, a greater condition advantage for sons than daughters has only been shown in one study on humans [9], and never in an industrialised population. To determine whether any sex ratio relationship is driven by success of resulting offspring in line with TWH [1], or simply occurs because sons cost more to raise [2], it is vital to determine if sons of mothers in good condition have higher reproductive success. However, in contemporary societies status can be negatively related to fertility [10]. Furthermore, most contemporary western societies have a monogamous social system, reducing differences in reproductive variance between sexes, although the change to monogamy appears to have been relatively recent [11].

Emerging evidence suggests that such an effect exists in mammals in relation to condition at conception [1], [12]. In humans, there has been extensive interest in sex ratio variation, but results have been inconsistent. While some studies in traditional societies support the TWH [e.g. 13], this is not so in industrialised humans [3], with most studies showing no difference [e.g. 14], [15], or small differences [e.g. 16]. Similar to studies in other mammals [12], studies on contemporary humans have used a variety of indices of condition with variable timing in the reproductive cycle, such as economic status [17], [18], female body shape [19] and size [20], [21], dominance, achievement or employment status [22], [23], and health [19]. In mammals, evidence suggests that sex ratios vary most consistently with condition around conception [12], [24], and physiological mechanisms may alter sex ratios during very early embryo development [12], [25], [26]. Recent evidence for sex ratio variation with diet at conception in humans [27] suggests similarity with other mammals. Furthermore, while the human birth sex ratio is male-biased [28] human in vitro raised embryos are more male-biased [e.g. 29], as in other mammal species [e.g.30] which has been attributed to glucose in the culture medium in cattle [25], [31]. Excess glucose kills female blastocysts but enhances male development [25]. However, alternate hypotheses in humans suggest that hormone levels [28], [32] or masculinity [22] are important regulators of human sex ratios. For example, levels of maternal testosterone are hypothesised to cause high-achieving women to give birth to more sons [23], [28]. Alternatively, relative ‘maleness’ in men has been proposed as an explanation for observed sex ratio biases by profession [22].

We examined whether the assumptions of the TWH, apply to the super-rich, using published information on family size among billionaires. This enabled us to distinguish between biases resulting from the costs [2] or the advantages [1] of raising sons. We then compared the offspring sex ratio of billionaires with the global population, and use information on these billionaires to test whether maternal condition influences sex ratios. Lastly, we test whether work achievement, as an index of testosterone levels [following 23], or paternal masculinity [22] explain variation in sex ratios.

## Materials and Methods

Information on US$ billionaires was extracted from the Forbes list (www.Forbes.com, 2008 list), including wealth, origin of wealth (self-made, inherited, and growing inheritance) residence and citizenship, and number of children (listed for 910 of 1046; 866 with at least one child; 71 female, 795 male). We then searched the billionaire's name online using Google, and used the resulting pages to determine the sex of children. Resulting pages included Wikipedia, bibliographic sites, company websites, and newspapers (particularly marriage, birth and death announcements). For sex ratio analysis we used those billionaires for whom we could ascertain the sex of every child (350 male billionaires, 49 female billionaires). We compared the sex ratio of billionaires with the population sex ratio using a chi-squared test. We then divided region of citizenship and residency into western Europe, eastern Europe, North America, South America, Asia, Africa, and Australasia, to control for cultural differences. Most billionaires were from monogamous societies, with fewer than 20 from Arab countries. Some Asian countries limit the number of children born, which could also influence results. We fitted a full model including interaction effects between gender and region of citizenship and gender and region of residency and the main effects, and used Akaike's Information Criterion [33] to select the most parsimonious model,. We square root transformed number of children to more closely approximate normality [34] and excluded two male billionaires that had grossly outlying number of offspring (37 and 61), since the rest of the population each had less than 20 children. We used a generalized linear logistic model with a logit link function [35] to test if offspring sex ratio differed with the source of wealth using the proportion of sons as the response variable, and gender and wealth-source as factorial predictors. Previous researchers have used work achievement as a proxy for testosterone levels [23]. We therefore reasoned that those billionaires who were either self-made or were growing their inheritance were high achieving (‘high work drive’), whereas those that had inherited their wealth were not as high achieving in employment (‘low work drive’). We fitted a model including all interaction effects and used AICs to select the minimal adequate model.

## Results

**Figure 1:**
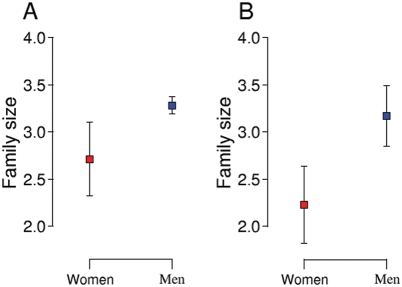
The sex bias in number of children born to billionaires by parental gender. (A) Number of children to female (71) and male (795) billionaires, and (B) the number of children to daughters and sons of an original billionaire (14), showing means±1 SE.

To test the assumptions of the TWH, we used only those billionaires that had children, and found that male billionaires had significantly more children and a more variable number of children than female billionaires (men: 1 to 61 children, women: 1 to 7 children; *F_1, 869_* = 4.36, *P* = 0.03; Fig. 1a). We identified 14 families where an original fortune had been made 2 generations ago, and where the fortune-maker (male in all cases) had at least one son and at least one daughter. This enabled us to trace the resulting grandchildren within a single family. The original fortune-maker left more grandchildren through his sons than through his daughters (mean of 1.33 more grandchildren, paired t-test: t_13_ = 3.09, P = 0.009; Fig. 1b). Furthermore, sons were, on average, richer than daughters within the same family (mean of 178 ranks higher on billionaire list; paired t-test; t_13_ = 2.45, P = 0.029), suggesting that sons received greater parental allocation. Therefore the assumptions of the model are met, and the TWH predicts that billionaires should give birth to more sons than daughters, and more sons than the non-billionaire population.

**Figure 2:**
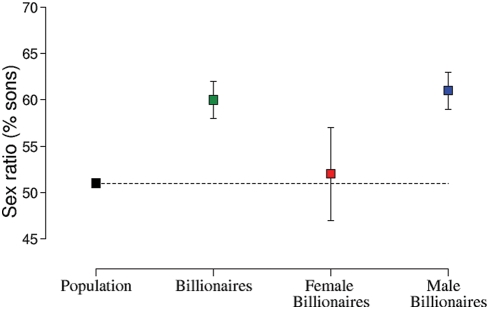
A comparison of sex ratios of children born to billionaires compared to the general population. Offspring sex ratio for the global population (Grant 1998), all billionaires for whom the sex of all children could be traced (399), as well as female billionaires (49) and females married to billionaires (350), showing means±1 SE.

Billionaires for whom we could locate information on all of their children had more sons than daughters, and significantly more sons than the general population (population: 51% sons, [28], billionaires 60% sons; *χ* ^2^ = 37.57, *DF* = 1, *P*<0.0001; Fig. 2). Therefore, mothers in the highest economic bracket had more sons than expected from the population average.

When we separated the billionaires by the sex of the listed billionaire we found that women who had children with billionaires had a more male biased offspring sex ratio than female who were themselves billionaires (*χ* ^2^ = 4.32, *DF* = 1, *P* = 0.04 Fig. 2). While the sex ratio of children to women who were themselves billionaires did not differ from the global population (53% male, *χ* ^2^ = 0.12, *DF* = 1, *P* = 0.73), women who had children with billionaires gave birth to significantly more sons (65% sons, *χ* ^2^ = 80.13, *DF* = 1, *P*<0.0001).

**Figure 3:**
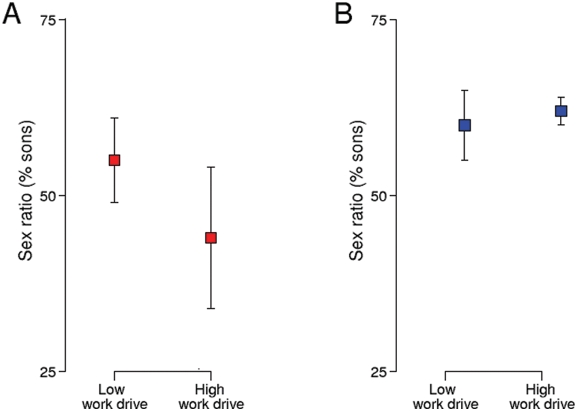
Offspring sex ratio for female billionaires and females married to billionaires in relation to work-drive or achievement where self-made and growing inheritance are considered high work drive as a proxy for high testosterone, showing means±1 SE.

The source and current status of wealth (inherited only vs self-made and growing inheritance) did not influence sex ratios of children fathered by billionaires (inherited only (60% sons) vs self-made (61% sons) and growing inheritance (65% sons) Fig. 3). However, among the women who were themselves billionaires, those that were self-made (52% sons) or were growing their inheritance (39% sons) had significantly fewer sons than those that inherited their wealth (56% sons, *χ* ^2^ = 4.32, *DF* = 1, *P* = 0.04, Fig 3).

## Discussion

Among all billionaires we show that male billionaires produce more children than female billionaires. Furthermore, when we investigated the grandchildren of an original fortune-maker, we found that he had more grandchildren through his sons than through his daughters, supporting the assumption that reproductive success would be enhanced by giving birth to more sons than daughters. Finally, parental allocation by the fortune-maker seemed to be higher into their sons than their daughters since sons ranked higher up the billionaire list than daughters. This is the first study to show that males have greater reproductive success in a sample from a contemporary, industrialized, monogamous society, and it supports previous research showing that high-income men have more children than either high-income women or low income men [36]. It also means that the assumptions of the TWH apply in this subset of society. Therefore, any differences in the production of sons and daughters are unlikely to be explained solely by differences in the cost of producing sons and daughters [2], [37], even though human sons seem to be more costly to produce [38]. Rather, our results suggest that there is a benefit to producing sons for billionaires [1]. In line with our predictions, billionaires gave birth to significantly more sons (60%) than the rest of the population (51%), and this was particularly marked for male billionaires (65% sons).

Hypotheses to explain biased sex ratios in humans include those relating levels of testosterone to offspring sex ratios [39], [32]. For example, Grant argues that maternal testosterone determines offspring sex ratios [28], and that testosterone levels are indicated by work achievement [23]. Other studies have confirmed a link between testosterone and work achievement [39], and people with entrepreneurial tendencies have higher levels of testosterone [40]. Contrary to expectations based on previous findings [23], women who were themselves billionaires, indicating high motivation, had fewer sons, and this was most marked among self-made women and those that were working and expanding their inheritance. Therefore, work achievement, was not a strong predictor of offspring sex ratios, suggesting that either maternal testosterone was not associated with sex ratio, or, more likely, that work achievement is a poor index of testosterone. Recent studies on other mammals have suggested that maternal testosterone levels are related to offspring sex ratios [41], [42]. However, theory also suggests that particularly high achieving women may have more influence over the future success of their daughters than their sons, or daughters may inherit aspects of their mother's rank [43], as seen in other primate species [44].

Previous studies have suggested a similar hormonal or masculinity effect in males, whereby high achieving males give birth to more sons, either because of hormone levels [32] or brain masculinity [22], both of which may be indexed by work achievement. However, there was no difference in sex ratio between inherited or high work-drive male billionaires, suggesting that male work achievement does not explain the observed sex ratio bias.

Finally, if sex ratio variation arose due to variation in maternal condition, we would predict that all mothers in the sample were in good condition, and no difference in sex ratio between the groups would be predicted. This was confirmed for all categories except the high-achieving billionairesses, although this may have been due to the small sample size in this category.

Therefore, contemporary humans in the highest economic bracket show a significant sex ratio bias in favour of sons as predicted by the TWH, and these sons leave more grandchildren for the parents than daughters. It seems likely that similar physiological mechanisms operate in human females as in other mammalian species [12], [26], since sex ratio variation seems to arise around conception in relation to maternal condition [27].
